# Different patterns of frontal and temporoparietal activities related to distinct inhibitory functions in adhd

**DOI:** 10.1192/j.eurpsy.2023.1281

**Published:** 2023-07-19

**Authors:** L. R. R. Carreiro, A. A. D. S. Afonso Junior, W. Machado-Pinheiro

**Affiliations:** 1 Postgraduate Program in Developmental Disorders, Mackenzie Presbyterian University, São Paulo; 2 Fluminense Federal University, Rio de Janeiro, Brazil

## Abstract

**Introduction:**

Inhibition is a core component of executive functions but is not a unitary construct. Instead, different inhibitory processes have specific behavioral effects and neural bases. Three important inhibitory functions explored by the literature are 1) interference control (i.e., inhibition of distractive information); 2) inhibition of prepotent responses and 3) inhibition of ongoing responses. These functions were described in the self-regulation theory as the possible main impairment in attention-deficit hyperactivity disorder (ADHD) and since then they have shown an association with several psychiatric disorders.

**Objectives:**

The current study investigated the neural bases of interference control, inhibition of prepotent responses, and inhibition of ongoing responses as they were assessed by a Stroop-matching/stop-signal task developed by our group.

**Methods:**

The Stroop-matching/stop-signal employs different conditions to create the demands for each inhibition which allows the assessment of these functions using a single protocol. Brain activations were acquired using fNRIS in a block-design method. The concentration of oxygenated hemoglobin (HbO). The first level analysis of HbO signals used a general linear model (GLM) to estimate individual brain activations. The second level analysis was performed using a linear mixed model to generate brain activations at the group level. Alpha level = 0.05 and the false discovery rate was applied when necessary. The sample was composed of 25 young adults (mean age = 21.8, SD = 4.39).

**Results:**

task Interference control showed activation in the left and right temporoparietal junction (TPJ), the right dorsolateral prefrontal cortex (DPLFC), and inferior frontal gyrus (IFG); inhibition of prepotent responses showed increased activity in the right IFG and left DLPFC; the suppression of ongoing responses showed a deactivation of the IFG and DLPFC bilaterally.

**Conclusions:**

These results indicate that the three inhibitory functions assessed present distinct brain patterns of function. The lateralization role was evident in DLPFC and IFG activities and recruitment of parietal areas seems to be limited to interference control in this protocol. Also, the stop-signal demand led to the deactivation of areas associated with the resolution of the primary Stroop-matching task. This study elucidates the role of brain mechanisms associated with specific inhibitory processes that are impaired in psychiatric disorders such as ADHD.

Financial support: FAPESP [grant 2019/20757-5, 2019/21773-4, 2020/14800-2]; CAPES Proex [grant 0426/2021, 23038.006837/2021-73]; Mackpesquisa; CNPq [grant 307443/2019-1]

**Disclosure of Interest:**

None Declared

